# Predicting a Need for Financial Assistance in Emergency Department Care

**DOI:** 10.3390/healthcare9050556

**Published:** 2021-05-10

**Authors:** Samuel Davis, Sara Nourazari, Rachel Granovsky, Nasser Fard

**Affiliations:** 1 Department of Mechanical and Industrial Engineering, Northeastern University, Boston, MA 02115, USA; davis.sam@northeastern.edu (S.D.); n.fard@northeastern.edu (N.F.); 2 Department of Health Care Administration, California State University, Long Beach, CA 90840, USA; 3 School of General Studies, Columbia University, New York, NY 10027, USA; rlg2179@columbia.edu

**Keywords:** healthcare finance, health equity, emergency department, predictive modeling, Medicaid

## Abstract

Identifying patients with a low likelihood of paying their bill serves the needs of patients and providers alike: aligning government programs with their target beneficiaries while minimizing patient frustration and reducing waste among emergency physicians by streamlining the billing process. The goal of this study was to predict the likelihood of patients paying the balance of their emergency department visit bill within 90 days of receipt. Three machine learning methodologies were applied to predict payment: logistic regression, decision tree, and random forest. Models were trained and performance was measured using 1,055,941 patients with non-zero balances across 27 EDs from 1 August 2015 to 31 July 2017. The decision tree accurately predicted 87% of unsuccessful payments, providing significant opportunities to identify patients in need of financial assistance.

## 1. Introduction

As hospitals and physician groups manage and recover from the COVID-19 pandemic, the importance of expanding our understanding of health finance to include the complex array of social and environmental determinants, the developing network of government programs, and the difficult tradeoffs between personal health and economic stability patients are faced with is evident.

Emergency physicians (EPs) treat all patients that present to the emergency department (ED) regardless of age, severity of injury, or their ability to pay. Due to this federal mandate, the ED has become the main source of primary care for many financially vulnerable patients. Of the 130 million ED visits which occurred in 2013, 36% were covered by private insurance [1]. However, patients often have less coverage than they expect, leaving them to face economic repercussions in addition to potentially significant health challenges. This is especially problematic for lower-income individuals who lack sufficient assets to cover their health insurance deductible [2].

The number of patients who are unable to pay for their healthcare bills is increasing, with hospitals providing upwards of USD 660 billion in uncompensated care since the turn of the century, with over USD 40 billion in 2019 alone [3]. If a patient has not paid the portion of the bill not covered by insurance, they are ultimately held personally responsible, despite a lack of resources, which may be compounded by a recent illness or injury that led to their unplanned emergency care.

Predicting patterns in payment behaviors would identify patients that would benefit from safety net resources and help providers to connect such qualifying patients with locally and federally funded services, such as Medicaid, grants, and social workers. These programs help patients avoid the financial burden of future medical bills and corresponding detrimental consequences, including the health risks of deferring necessary care due to financial concerns [4,5]. At a systems level, this can also serve to reduce fragmented care.

Relatively few studies address the prediction of payment behavior and the significant burden created by unpaid healthcare bills. In 1991, Zollinger used a multiple regression model to determine the effect of patient and hospital characteristics on the variation in the amount of hospital charges left unpaid across 28 Indiana hospitals. He found that insurance coverage, total hospital charge, pregnancy, marital and employment status, urban location, and total hospital revenue were significant factors in predicting unpaid hospital bills, while length-of-stay, gender, age, and diagnoses unrelated to pregnancy and childbirth were not significant. Notably, 60% of patients with uncompensated balances had some form of health insurance but were responsible for 40% of the amount owed, emphasizing the need to increase both health care coverage and collection efforts [6]. The finding that increasing health insurance coverage decreases uncompensated care is supported by the sweeping reduction in the burden of uncompensated care throughout states that expanded Medicaid under the Affordable Care Act (ACA), with estimated savings of USD 6.2 billion across hospitals in Medicaid expansion states between 2013 and 2015 [7].

Several other studies looked at bad debt versus charity care, in which the exact write-off is carefully provided via a systematic process [8,9,10]. Weissman importantly differentiated between bad debt that created personal patient liability and balances addressed through charity care, analyzing the characteristics of patients that remained responsible for additional balances across six hospitals in Massachusetts in 1992, finding that while most healthcare debt was not able to be satisfied by the patient (58%), charity care represented nearly two-thirds of the total write-off amount (63%). In addition, 73% of the patients faced with outstanding balances were uninsured compared to only 50% of the charity cases, with pregnant women and newborns representing the largest source of uncompensated care write-offs [11].

In recent years, healthcare systems have readily applied various data mining techniques to address a wide variety of issues, ranging from determining effective treatment protocols to detecting insurance fraud [12,13]. This new technology has also been utilized to model patterns of patient healthcare debt.

In 2005, Zurada and Lonial compared the efficacy of five data mining techniques in evaluating whether patients are likely to have the means to reduce their healthcare debt, in an attempt to effectively understand and predict payment behaviors without the insight of a patient’s financial information. Neural networks, decision trees, logistic regression, memory-based reasoning, as well as the ensemble method (a combination of the first three methods) were applied to a dataset of several thousand patients, examining four characteristics (age, gender, diagnosis, and the total amount owed) to predict whether the patient would be able to repay their debt burden. Logistic regressions, neural networks, and the ensemble method exhibited the most accurate classifications, with the neural network model classifying the most unpursued cases as likely to pay, providing a potential source of additional recovered income [8].

Four recent works have investigated the effectiveness of computational intelligence methods to the task of predicting patients’ ability to decrease their healthcare debt in imbalanced datasets, showing support for the role of cost-sensitive learning methods in the classification of unknown cases, as well as Bayesian network-based models [9,10,11,14].

The objectives of this study were to develop and compare the ability of machine learning (ML) models to predict successful ED visit payment accurately, to help identify patients in need of financial support, and to lay the foundation for programs that support patients obtaining access to available low-cost healthcare services. This study expands existing work by using a significantly larger dataset, both in the number of patients and features.

## 2. Materials and Methods

Using data provided by a national billing company, a retrospective, cross-sectional study was conducted using two years of de-identified patient billing records from 1 August 2015 to 31 July 2017. The dataset included 1,055,941 patients with positive balances due from 27 blinded EDs across 13 states. The available data were a combination of information gathered at patient registration and after billing the patient’s health insurer. Of these patients, 39% (412,209) successfully paid their bills within 90 days. The remaining patients did not close their balance in 90 days. The sites and commercial insurance carriers were blinded to protect the site identities.

Twelve features were used to model and predict successful payment, as shown in Table 1. For classification and prediction purposes, paid bills were labeled as “positive”, while unpaid bills were labeled as “negative”. The first feature represents if the patient was seen by just a physician, as opposed to a shared visit with an advanced practice provider. Three features represent the patient’s gender, age, and out-of-state residency. The ages were grouped as: under 18, 18–24, 25–34, 35–44, 45–54, 55–64, 65–79, and over 80, with the variable representing ages 35–44 omitted and used as a base as it contained the median age. Additional features represented whether the patient provided a work phone number and the primary insurance grouped as Medicare, Medicaid, Private, Other, and None, with Private serving as the base. Notably, this was the insurance information gathered at registration, which in some cases did not represent the true payer.

A binary feature was created indicating if the patient had secondary insurance or not, and another feature categorized if the patient was the policyholder, or their spouse, or some other relationship. The patient’s marital status was categorized as single, married, or in another marital status, while the patient’s debt burden was broken into five tiers: under USD 25, USD 25–100, USD 100–300, USD 300–600, and over USD 600, with the USD 100 to USD 300 group omitted and used as a base as it contained the median debt burden. The primary current procedural terminology (CPT) code signified four visit level severities (99283, 99284, 99285, and 99291), and “all other” codes—99284 was set as the base, representing an average ED visit severity, while binary variables were created for the four remaining levels.

We developed and tested three ML models to predict payment behavior: logistic regression (LR), decision tree (DT), and random forest (RF). Similar to linear regression, LR is a statistical technique that determines the optimal set of coefficients to multiply by each independent factor such that the sum of squared error is minimized. LR then transforms the output to a probability, ranging from 0 to 1. In general, if the likelihood is greater than 50%, then the model predicts a positive outcome, and negative otherwise. Alternatively, DT uses recursive partitioning to split a dataset into classes, one field at a time. At every stage, each threshold within its respective field is examined to determine the optimal split, such that the two resulting datasets are the most pure (i.e., tending towards the positive or negative outcome). RF expands upon traditional DTs. Instead of making a single tree, many trees are created, each programmed to be slightly different due to random feature selection. When predicting an outcome, each tree gets a “vote” on the outcome, with majority rules. Data were prepared in Microsoft Excel, and statistical analyses were performed using the R statistical programming language.

**Figure 1:**
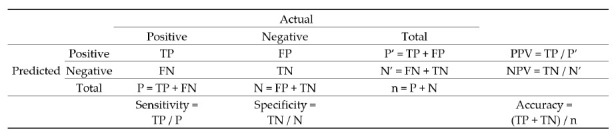
Confusion Matrix. Correct predictions are either TP or TN, while incorrect predictions are either FP or FN. Sensitivity is the ratio of TP to all P, which represents the strength of the model at identifying positive results. The specificity is the ratio of TN to all N, which represents the strength of the model at identifying negative results. The PPV represents the likelihood of a positive prediction being correct, while NPV represents the likelihood of a negative prediction being correct.

To develop each predictive model, 80% of the full dataset was first used to “train” the model, leaving 20% to serve as the test dataset to assess model performance. This training protocol reduces bias when assessing the quality of each model. Each record in the test dataset was placed into one of four buckets: true positive (TP), true negative (TN), false positive (FP), and false negative (FN). This bucketing is based on whether the true result is positive (P) or negative (N), and if the model predicts positive (P’) or predicts negative (N’). In addition to accuracy, we also measured sensitivity, specificity, positive predictive value (PPV), and negative predictive value (NPV). Overall accuracy is the ratio of correct predictions to the total number of records (n). The confusion matrix in Figure 1 shows the equations for all five performance measures.

Prior to running the ML algorithms, we applied the chi-squared test for homogeneity to each factor. The primary hypothesis is as follows:

**Hypothesis** **H1.** *The frequency of paid bills has no relation to the level of each factor.*

**Hypothesis** **H2.** *The frequency of paid bills is related to the level of each factor.*

## 3. Results

All features, except for the variable indicating whether a work phone was provided, were statistically significant (*p* < 0.001), for which we reject the null hypothesis. We found that higher severity cases, indicated by CPT level and procedures, were more likely to fully resolve the debt burden, as well as older, female, and out-of-state patients. Spouses, children, and married patients were also more likely to successfully pay. Patients with Medicare and private insurance, secondary insurance, and smaller bills had a higher likelihood of payment. Table 2 indicates the percentage of patients successfully paying for all features.

While the factor analysis looked at each variable independently, the LR holistically modeled all variables simultaneously and produced an odds ratio (OR) instead of a probability. The OR is the ratio of the probability of success to the probability of failure. For instance, the OR of 1.97 for ages 0–17 means that in the absence of other information, patients under seventeen years of age have almost twice the odds of a successful payment relative to a patient having all the base levels. The OR increases substantially with both ages above 35–44 and below. Patients without insurance represent the lowest OR out of all variables. Spouses and children were more likely to pay, as were married adults. Like the factor analysis, smaller bills were also correlated with a higher likelihood of successful payment. Table 3 shows the results of the LR analysis, including all ORs, confidence intervals, and *p*-values.

**Figure 2:**
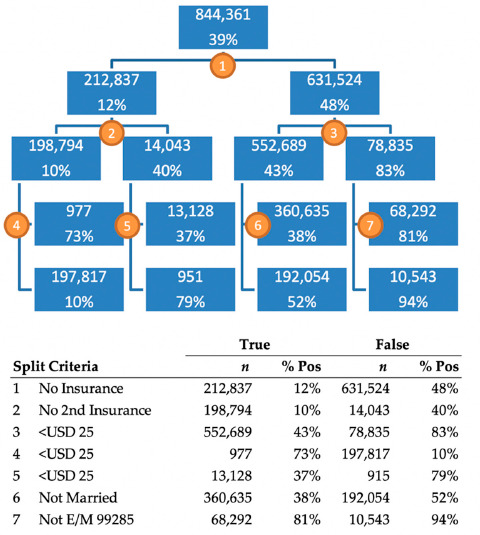
Abridged DT results; the complete tree has 131 nodes, of which 66 are terminal.

Although the full DT includes 131 nodes, which is too large to display, the first four levels are shown in Figure 2. The top value in each node represents the number of patients in the training dataset, and the bottom value represents the proportion of patients that successfully paid their bill. The first split of this tree was made on the level representing patients with no insurance. The 212,837 patients for which this was true had a 12% payment rate and the 631,524 that did not, had a 48% payment rate. The count of 212,837 patients differs from the count of 266,029 patients in Table 2 because this DT used only the training data, which made up 80% of the total dataset. The DT shows us that the most important indicator in the dataset is if a patient does not have insurance. If they do have insurance, the next most important variable is if the amount owed is under USD 25. The tree indicates that patients with insurance, owing less than USD 25, and a high severity case, paid 94% of the time, while patients without primary or secondary insurance who owed more than USD 25 only paid 10% of the time.

The three ML models and five performance metrics are shown in Table 4. RF performed best on all measures except for specificity, for which DT performed best. The models were similar in scores, without a substantial range in performance. Notably, all models had substantially higher specificity versus sensitivity, which indicates that unsuccessful payments were predicted with higher accuracy than successful payments. This pattern suggests there is still much more to be understood regarding why some patients are unable to pay their bills.

## 4. Discussion

Developing a patient-oriented payment process in healthcare operations and finance is one of the areas that has not been well-studied or investigated. The objective of this work is to highlight this gap and provide an opportunity for forming data-informed strategies to support patients and help strengthen healthcare systems’ financial resiliency. We aimed to develop and compare ML models to better predict which patients may need assistance in reducing the debt burden of their ED bills to help hospitals identify and connect patients in need of additional resources. Computing the likelihood of successful payments can help a practice to connect with patients more effectively and minimize system-wide inefficiencies.

Our results indicate that patients with larger financial responsibilities, especially single young adults, are the most likely patients to struggle with paying their ED bills. Most significant is that patients without insurance are not able to fully resolve their debt 88% of the time. This result highlights the critical gap in the American healthcare system, where the same patients who do not have the financial resources to seek preventative care must rely on the compassion of EPs, and subsequently face large bills which put them further in economic distress. The findings in this work concur with previous studies examining the reinforcing cycle of inadequate health coverage [6,8,9,10,11,12,13,14].

Our approach provides a holistic view of the systemic inability of many patients to pay for healthcare services. Individuals who lack health insurance often end up relying on EDs for necessary treatment and face large, unexpected medical bills later which they do not have the financial capabilities to pay, creating a reinforcing cycle of worsening credit and economic stress. By identifying patients who are likely to have difficulty paying medical bills in advance, EDs can effectively target them with intervention strategies meant to reduce their financial burden, ranging from early, transparent communication of costs to flexible or simplified payment options, or enrollment in local or federally funded programs. Furthermore, the likelihood of successful payments can be used to organize patient outreach efficiently: instead of trying to maximize accuracy across all patients, the algorithms can be adjusted to maximize the relative weighted value of identifying positives or negatives depending on the interest of the user.

At a macro level, this approach can also help investigate which social determinants of health are more pronounced in a specific patient population, triggering frequent utilization of ED medicine rather than establishing a primary care physician, which would not only improve the patient’s health at the individual level but also positively contribute to improved population health. This can also help inform policymakers and large purchasers on resource allocations and support for nationally and state-funded patient navigation programs, allowing for developing customized and patient-oriented payment plans. By applying predictive modeling approaches, hospitals can take a proactive role in helping patients to benefit from state-funded programs and navigate through the network restrictions, out-of-pocket costs, and confusion surrounding healthcare costs and billing systems.

Solutions such as those proposed in this study can help ACA navigators or advisors residing at hospitals or health systems to provide patients a clear picture of the insurance options available to them. They can also help individuals or families apply for affordable health insurance or state-funded programs and help them overcome obstacles to enrollment. This assistance can be crucial for patients and healthcare systems as new Medicaid enrollees can receive retroactive coverage for care received in the past 90 days in most states. Furthermore, having health insurance coverage is known to increase the likelihood that a patient will seek needed follow-up and preventative care, which at a systems level can reduce healthcare costs and improve population health.

A potential limitation of this study is that all patient data were aggregated and de-identified, and thus lacked potentially important information such as zip-code, visit frequency, and payment history. This information would be readily available for hospitals, social workers, and navigators, and can be incorporated in the utilized predictive models. In addition, the insurance information was as collected, and not necessarily the true insurance. As the billing cycle proceeds, it is not uncommon for a patient’s initial insurance information to differ from the carrier eventually billed. This is a potentially confounding variable in our analysis.

Future work should further explore the most effective methods to help patients who are struggling financially, as well as applying the ML models to other ED processes. The integration of predictive models into the triage process could help to predict patients that might leave without being seen, patients who could potentially be admitted to a higher level of care, and patients who are likely to return within 72 h. Real-time data-driven operations would support proactive patient outreach, bed management, and service recovery.

In summary, we achieved 72.4% accuracy in predicting successful ED payment by applying three ML algorithms, thus highlighting those remaining patients in need of additional resources. Patients with larger bills, no insurance, and young adults were at the highest risk of facing significant healthcare debt burden which they were unable to resolve.

## Figures and Tables

**Table 1:** Twelve categorial features used to model payment results.

#	Feature	Grouping
1	Physician Only	Yes, No
2	Primary CPT	99283, 99284, 99285, 99291, “all other” codes
3	# of CPT codes	1, 2+
4	Patient Age	0–17, 18–24, 25–34, 35–44, 45–54, 55–64, 65–79, 80+
5	Gender	Female, Male
6	Out of state	No, Yes
7	Work phone	No, Yes
8	Primary insurance	Medicare, Medicaid, Private, Other
9	Secondary insurance	No, Yes
10	Relationship	Self, Spouse, Other
11	Marital status	Single, Married, Other
12	Patient responsibility	Under USD 25, USD 25–100, USD 100–300, USD 300–600, USD 600+

**Table 2:** Feature analysis showing the proportion paid for each level and chi-square test results for each feature.

Characteristic	Paid *n* = 412,209	Unpaid *n* = 643,732	Total *n* = 1,055,941	Paid %	Chi-Square Statistic	*p*-Value
				39.0%		
Physician						
Y	299,037	420,085	719,122	41.6%	6143.1	<0.001
N	113,172	223,647	336,819	33.6%		
Primary CPT						
99283	88,734	168,796	257,530	34.5%	4201.4	<0.001
99284	144,804	229,637	374,441	38.7%		
99285	149,148	208,494	357,642	41.7%		
99291	16,327	20,699	37,026	44.1%		
Other	13,196	16,106	29,302	45.0%		
# of CPT Codes						
1	250,777	457,637	708,414	35.4%	11,966.4	<0.001
2+	161,432	186,095	347,527	46.5%		
Patient Age						
0–17	49,604	64,075	113,679	43.6%	93,785.6	<0.001
18–24	38,064	96,132	134,196	28.4%		
25–34	49,603	150,315	199,918	24.8%		
35–44	47,077	117,222	164,299	28.7%		
45–54	60,400	103,120	163,520	36.9%		
55–64	71,525	68,523	140,048	51.1%		
65–79	60,764	34,084	94,848	64.1%		
80+	35,172	10,261	45,433	77.4%		
Gender						
Female	217,199	315,202	532,401	40.8%	1396.1	<0.001
Male	195,010	328,530	523,540	37.2%		
Out of State						
N	367,791	587,716	955,507	38.5%	1255.7	<0.001
Y	44,418	56,016	100,434	44.2%		
Work Phone						
N	353,854	552,261	906,115	39.1%	0.6	0.635
Y	58,355	91,471	149,826	38.9%		
Primary Insurance						
Medicare	107,134	69,267	176,401	60.7%	132,260.1	<0.001
Medicaid	25,541	45,450	70,991	36.0%		
Private	232,763	279,271	512,034	45.5%		
Other	21,662	26,278	47,940	45.2%		
None	25,109	223,466	248,575	10.1%		
Secondary Insurance						
N	319,321	585,117	904,438	35.3%	36,874.9	<0.001
Y	92,888	58,615	151,503	61.3%		
Relationship						
Self	332,186	573,757	905,943	36.7%	15,370.9	<0.001
Spouse	34,364	26,924	61,288	56.1%		
Other	45,659	43,051	88,710	51.5%		
Marital Status						
Single	187,185	396,415	583,600	32.1%	28,347.0	<0.001
Married	165,646	168,597	334,243	49.6%		
Other	59,378	78,720	138,098	43.0%		
Patient Responsibility						
<USD 25	83,229	17,508	100,737	82.6%	113,249.8	<0.001
USD 25–100	104,811	110,896	215,707	48.6%		
USD 100–300	80,917	166,145	247,062	32.8%		
USD 300–600	81,676	199,839	281,515	29.0%		
>USD 600	61,576	149,344	210,920	29.2%		

**Table 3:** LR results, including odds ratio (OR), confidence interval (CI), and *p*-value for each non-base level by feature.

Characteristic	OR	CI (95%)	*p*-Value	Characteristic	OR	CI (95%)	*p*-Value
Intercept	0.36	(0.35–0.37)	<0.001	Work phone			
Physician				N	base	base	base
Y	base	base	base	Y	1.17	(1.16–1.19)	<0.001
N	0.90	(0.89–0.91)	<0.001	Primary Insurance			
Primary CPT				Medicare	0.49	(0.48–0.50)	<0.001
99283	0.99	(0.97–1.00)	0.146	Medicaid	0.91	(0.89–0.93)	<0.001
99284	base	base	base	Private	base	base	base
99285	1.08	(1.07–1.10)	<0.001	Other	1.02	(0.99–1.05)	0.131
99291	1.00	(0.97–1.03)	0.858	None	0.23	(0.22–0.23)	<0.001
Other	1.13	(1.09–1.16)	<0.001	Secondary Insurance			
# of CPT Codes				N	base	base	base
1	base	base	base	Y	1.66	(1.64–1.69)	<0.001
2+	1.00	(0.99–1.02)	0.580	Relationship			
Patient Age				Self	base	base	base
0–17	1.97	(1.92–2.01)	<0.001	Spouse	1.22	(1.20–1.25)	<0.001
18–24	1.15	(1.12–1.17)	<0.001	Other	1.36	(1.34–1.39)	<0.001
25–34	0.97	(0.95–0.99)	<0.001	Marital Status			
35–44	base	base	base	Single	base	base	base
45–54	1.30	(1.28–1.33)	<0.001	Married	1.61	(1.59–1.63)	<0.001
55–64	2.16	(2.12–2.20)	<0.001	Other	0.87	(0.85–0.88)	<0.001
65–79	3.53	(3.45–3.62)	<0.001	Patient Responsibility			
80+	7.64	(7.39–7.90)	<0.001	<USD 25	7.56	(7.39–7.74)	<0.001
Gender				USD 25–100	1.18	(1.17–1.20)	<0.001
Female	base	base	base	USD 100–300	base	base	base
Male	0.99	(0.98–1.00)	0.004	USD 300–600	1.06	(1.04–1.07)	<0.001
Out of State				>USD 600	1.01	(1.00–1.03)	0.138
N	base	base	base				
Y	1.34	(1.32–1.36)	<0.001				

**Table 4:** Overall model results. RF performed the best in all metrics except specificity, where DT performed best.

Experiment	Accuracy	Sensitivity	Specificity	PPV	NPV
**LR**	71.3%	49.7%	85.3%	68.6%	72.4%
**DT**	71.7%	47.9%	87.2%	70.7%	72.1%
**RF**	72.4%	49.9%	86.9%	71.1%	72.9%

## Data Availability

Data sharing not applicable.
